# Distinct Roles of Classical and Lectin Pathways of Complement in Preeclamptic Placentae

**DOI:** 10.3389/fimmu.2022.882298

**Published:** 2022-05-31

**Authors:** Beatrice Belmonte, Alessandro Mangogna, Alessandro Gulino, Valeria Cancila, Gaia Morello, Chiara Agostinis, Roberta Bulla, Giuseppe Ricci, Filippo Fraggetta, Marina Botto, Peter Garred, Francesco Tedesco

**Affiliations:** ^1^ Tumor Immunology Unit, Department of Health Promotion, Mother and Child Care, Internal Medicine and Medical Specialties “G. D’Alessandro”, University of Palermo, Palermo, Italy; ^2^ Pathology Unit, Azienda Sanitaria Provinciale (ASP) Catania, “Gravina” Hospital, Caltagirone, Italy; ^3^ Institute for Maternal and Child Health, IRCCS Burlo Garofolo, Trieste, Italy; ^4^ Department of Life Sciences, University of Trieste, Trieste, Italy; ^5^ Department of Medical, Surgical and Health Science, University of Trieste, Trieste, Italy; ^6^ Department of Immunology and Inflammation, Imperial College London, London, United Kingdom; ^7^ Imperial Lupus Centre, Imperial College Healthcare National Health Service (NHS) Trust, London, United Kingdom; ^8^ Laboratory of Molecular Medicine, Department of Clinical Immunology, Copenhagen University Hospital, Rigshospitalet, Copenhagen, Denmark; ^9^ Department of Clinical Medicine, University of Copenhagen, Copenhagen, Denmark; ^10^ Istituto Auxologico Italiano, Laboratory of Immuno-Rheumatology, IRCCS, Milan, Italy

**Keywords:** complement system, pre-eclampsia, vascular remodeling, C1q, ficolin-3

## Abstract

Pre-eclampsia is a pregnancy complication characterized by defective vascular remodeling in maternal decidua responsible for reduced blood flow leading to functional and structural alterations in the placenta. We have investigated the contribution of the complement system to decidual vascular changes and showed that trophoblasts surrounding unremodeled vessels prevalent in preeclamptic decidua fail to express C1q that are clearly detected in cells around remodeled vessels predominant in control placenta. The critical role of C1q is supported by the finding that decidual trophoblasts of female *C1qa^-/-^* pregnant mice mated to *C1qa^+/+^* male mice surrounding remodeled vessels express C1q of paternal origin. Unlike *C1qa^-/-^* pregnant mice, heterozygous *C1qa^+/-^* and wild type pregnant mice share a high percentage of remodeled vessels. C1q was also found in decidual vessels and stroma of normal placentae and the staining was stronger in preeclamptic placentae. Failure to detect placental deposition of C1r and C1s associated with C1q rules out complement activation through the classical pathway. Conversely, the intense staining of decidual endothelial cells and villous trophoblast for ficolin-3, MASP-1 and MASP-2 supports the activation of the lectin pathway that proceeds with the cleavage of C4 and C3 and the assembly of the terminal complex. These data extend to humans our previous findings of complement activation through the lectin pathway in an animal model of pre-eclampsia and provide evidence for an important contribution of C1q in decidual vascular remodeling.

## Introduction

Pre-eclampsia (PE) is a serious clinical condition that occurs in 3-5% of pregnant women in the second half of pregnancy after 20 weeks and more frequently after 34 weeks of gestation and is characterized by hypertension, proteinuria, liver and cerebral involvement (1). The disorder is responsible for maternal morbidity and mortality and adverse pregnancy outcomes, including preterm delivery, intrauterine growth retardation and fetal death (2). The diverse clinical presentations of PE argue for a multifactorial nature of this syndrome whose pathogenesis has not yet been clearly defined despite the efforts made over recent years to identify the possible causes.

As the clinical signs disappear with the termination of pregnancy after placental expulsion, the placenta has been recognized to play a major role in the development of PE. This newly formed organ at the feto-maternal interface undergoes structural and functional changes that start with defective remodeling of decidual spiral arteries, particularly evident in the early-onset PE (3, 4), and endothelial dysfunction (5, 6) resulting in high resistance vessels, reduced tissue perfusion and oxidative stress (7).

These changes stimulate an inflammatory response in the second stage of PE involving both cells and soluble molecules of the innate immune system (8, 9).

Complement (C) is a critical component of innate immunity and has been implicated in the development of PE. The system plays an important role in host defense and homeostasis following activation *via* the classical, the lectin and the alternative pathways, but it may also cause tissue damage under conditions of unrestricted activation (10–12). While the alternative pathway functions as an amplification loop for the classical and lectin pathways, the activation of latter two pathways requires the intervention of recognition molecules to initiate the triggering process. C1q serves this function for the classical pathway and acts in association with the serine proteases C1r and C1s. Conversely, the lectin pathway is triggered by a set of different recognition molecules belonging to the collectin family (mannose-binding lectin aka MBL, collectin-10 aka CL-10 and collectin-11 aka CL-11) and the ficolin family (ficolin-1, ficolin-2, and ficolin-3). All the recognition molecules of the lectin pathway are found associated with a set of serine proteases named MASPs (MASP-1, MASP-2 and MASP-3) and regulatory molecules named MAPs (MAP-1 and MAP-2) (13). Activation of the classical and lectin pathways leads to the enzymatic cleavage of C4, C3 and C5, and eventually results in the release of potent anaphylatoxins, C3a and C5a, and the assembly of the C5b-9 complex. Evidence has been collected over the years suggesting an involvement of C in placental alterations of PE (14, 15). Increased levels of C3a, C5a and the soluble sC5b-9 complex have been reported in the circulation of preeclamptic patients (16, 17) and in the urine of patients with severe eclampsia suggesting their contribution to C-mediated renal damage (18). The finding of high levels of Bb, a split product of the C factor B of the alternative pathway, in the early phase of gestation in women who later developed PE led Lynch and colleagues to propose Bb as an early biomarker for the development of PE (19). The observation, however, was not confirmed in a prospective study performed in Caucasian patients (16). These conflicting results may be due to racial differences in the patients studied since the levels of Bb were found to be significantly higher in African-American PE patients (20).

Immunohistochemical analysis of PE placenta revealed increased deposition of C components and C activation products in chorionic villi compared to control placentae (21–25). Diffuse C4 staining has been found to be associated with placental pathologic changes and fetal growth restriction, and has been proposed as biomarker for adverse pregnancy outcomes in PE patients (23, 25). However, the pathway of C activation and the mechanisms involved in C-mediated tissue damage have not been fully elucidated. More direct evidence for C involvement in PE has been obtained in an animal model where CBA/J female mice mated to DBA/2 males have high resorption rate and fetal growth retardation (26). The pregnant mice manifest features common to PE, including albuminuria, endotheliosis and increased sensitivity to angiotensin II that correlates with adverse pregnancy outcomes (27). The fetal loss in the CBA/J female mice can be prevented by administering either an inhibitor of the C3 convertase, Crry-Ig, or a neutralizing anti-C5 monoclonal antibody or a C5a receptor antagonist (28). The interesting observation by Singh *et al.* that pregnant C1q-deficient mice present the classical manifestations seen in PE patients, including hypertension, albuminuria, and increased levels of soluble VEGF receptor 1 (sFlt-1), suggests that C1q may exert a surprising protective role in pregnancy (29). In this respect, it is important to note that C1q is widely distributed in maternal decidua in normal pregnancy and is locally synthesized and secreted by macrophages as well as endothelial cells and extravillous trophoblasts (30, 31). Considering the controversial findings in the literature herein we aimed to investigate the contribution of C1q expressed by perivascular trophoblasts to the physiologic process of decidual vascular remodeling and to explore the impact that a defect in the local expression of C1q may have on the vascular changes. In addition, we sought to determine whether locally expressed C1q is involved in the activation of the classical pathway at placental site, and whether C activation may also be triggered *via* the lectin pathway as observed in an animal model of PE (32).

## Materials and Methods

### Study Groups

Fifteen women with pregnancies complicated by early-onset PE that developed before 34 weeks of gestation and a control group of 15 healthy women with uncomplicated pregnancies were included in this investigation. Preeclampsia was defined as newly diagnosed high blood pressure (systolic blood pressure ≥140 mmHg or diastolic blood pressure ≥90 mmHg) with onset after 20 weeks of gestation and proteinuria (24-hour urine protein ≥300 mg or a dipstick ≥1+) measured on two or more occasions at 6-8 hour intervals (33). IUGR was observed in 7 out 15 (47%) PE patients.The clinical characteristics of the study groups are shown in **Table 1**. Patients and control pregnant women were enrolled at the Institute for Maternal and Child Health, IRCCS Burlo Garofolo, Trieste, Italy. The study was reviewed and approved by the Regional Ethical Committee of FVG (CEUR), Udine, Italy (CEUR-2020-Os-156; Prot. 0022668/P/GEN/ARCS).

**Table 1:** Clinical characteristics of women enrolled in the study.

Characteristics	Pre-eclampsia (n = 15)	Controls (n = 15)
Mean maternal age, y (SD)	35.1 ( ± 4)	33.5 ( ± 2)
Mean maternal BMI, kg/m^2^ (SD)	25 ( ± 3)	19.5 ( ± 5)*
Nulliparity (%)	60%	40%
Highest diastole, mm Hg (SD)	89 ( ± 15)	66 ( ± 10)
Highest systole, mm Hg (SD)	141 ( ± 14)	109 ( ± 13)
Proteinuria, g/24 h (SD)	1996 ( ± 1202)	ND
Gestational age at delivery, weeks+ days (min-max in weeks)	29+3 (25–33)	39+4 (38–42)**
Birth weight, g (SD)	1040 ( ± 448)	3285 ( ± 276)**
Placenta weight, g (SD)	301 ( ± 78)	560 ( ± 58)*
**Mode of delivery**		
Cesarean section (%)	60%	20%

SD, standard deviation. ND, not detectable by urine dipstick test. *p < 0.001; **p < 0.0001 (T-Student test).

### Human Placental Tissues

Three to four biopsy samples were taken from the central area of the maternal surface of placentae collected immediately after delivery and quickly washed in saline to remove residual blood. The specimens were fixed in 10% buffered formalin and paraffin-embedded. Informed consent was obtained from all participants in the study.

### Mice

C57BL/6 mice were purchased from Harlan Laboratories. C1q deficient mice (*C1qa^-/-^*) backcrossed on the C57BL/6 background were generated as previously described (34). Wild Type (WT) C57BL/6 males were mated with *C1qa^-/-^* females to generate heterozygous mice. Implantation sites were collected from pregnant mice on days 13-14, fixed in 10% buffered formalin and embedded in paraffin. All animals were handled in accordance with the institutional guidelines and in compliance with the European (86/609/EEC). The UK Home Office approved the procedure.

### Histochemical, Immunohistochemical and Immunofluorescence Analysis

Four micrometers-thick serial sections were cut from deparaffinized and rehydrated human and mouse placental samples. Some sections were stained with hematoxylin-and-eosin and examined for the distribution of invasive extravillous trophoblast and the structure of decidual blood vessels.

Deposits of C components and C activation products were analyzed on adjacent sections immunostained after antigen retrieval using Novocastra Epitope Retrieval Solution at pH6, pH8 or pH 9 to unmask antigens in a thermostatic bath at 98°C for 30 min. Subsequently, the sections were brought to room temperature and washed in phosphate buffered saline (PBS). After neutralization of the endogenous peroxidases with 3% H_2_O_2_ and Fc-blocking by 0.4% casein in PBS (Novocastra), the sections were incubated with the primary antibodies listed in **Table 2**. Liver sections were used as positive controls for MBL, ficolin-2, C1r and C1s staining (**Supplemental Figure 1**).

**Table 2:** Antibody sources and dilutions.

Antigens	Reactivity	Dilution	Type	Code number	Source
C1q	human	1:500	rabbit pAb	A0136	Agilent, DK
C1r	human	1:100	rabbit pAb	HPA001551	Sigma Aldrich
C1s	human	1:250	goat pAb	A302	Quidel
Ficolin-1	human	1:50	mouse mAb	FCN166	(35)
Ficolin-2	human	1:500	mouse mAb	FCN219	(36)
Ficolin-3	human	1:1000	mouse mAb	FCN 309	(37)
MBL	human	1:1000	rabbit pAb	HPA002027	Sigma Aldrich
MASP-1	human	1:40	rabbit pAb	HPA001617	Sigma Aldrich
MASP-2	human	1:20	rabbit pAb	HPA029313	Sigma Aldrich
C3d	human	1:100	rabbit pAb	403A-76	Cell Marque
C4d	human	1:100	rabbit pAb	404A-16	Cell Marque
C9neo	human	1:50	mouse mAb	HM2264-IA	Hycult Biotech
CK-7	human	1:8000	rabbit mAb	Ab181598	Abcam
α-SMA	human	1:500	mouse mAb	SKU001	BioCare
C1q	mouse	1:400	rabbit pAb	NA	(38)
CK-7	mouse	1:8000	rabbit mAb	Ab181598	Abcam
CD31	mouse	1:50	rabbit pAb	Ab28364	Abcam

pAb, polyclonal antibody; mAb, monoclonal antibody; CK, cytokeratin; α-SMA, α- smooth muscle actin; NA, not available.

For multiple-marker immunostaining, the sections were subjected to sequential rounds of single-marker immunostaining, and the binding of the primary antibodies was revealed using specific secondary antibodies conjugated with different enzymes or fluorophores.

Immunohistochemical staining was developed using the Novolink Polymer Detection Systems (Novocastra) or IgG (H&L)-specific secondary antibodies (Life Technologies, 1:500) and AEC (3-Amino-9-ethylcarbazole) or DAB (3,3’-diaminobenzidine) as substrate chromogens. Double immunohistochemistry (IHC) was performed by applying Signal Stain Boost IHC Detection (Cell Signaling) alkaline phosphatase-conjugated and Vulcan Fast Red as substrate chromogen.

The Opal Multiplex IHC kit (Akoya Biosciences) was used to stain the tissue sections with antibodies raised in the same species. After deparaffinization, antigen retrieval in pH9 buffer was brought to a boil at 100% power, followed by 20% power for 15 minutes using microwave technology. The sections were treated with blocking buffer for 10 minutes at room temperature before incubation with the primary antibody. The slides were then incubated with polymeric horseradish peroxidase-conjugated (HRP) secondary antibody for 10 minutes and the signal was visualized using Opal 520 fluorophore-conjugated tyramide signal amplification (TSA) at 1:100 dilution. The HRP catalyzes covalent deposition of fluorophores around the marker of interest. The slides were again processed with the microwave treatment to strip primary/secondary antibody complex and allow the next antigen-antibody staining. Another round of staining was performed with the second primary antibody incubation, followed by HRP secondary antibody and Opal 620 fluorophore-conjugated TSA at 1:100 dilution for signal visualization. Finally, the slides were again microwaved in antigen retrieval buffer and nuclei were subsequently visualized with DAPI (4’,6-diamidin-2-fenilindolo). The slides were analyzed under a Zeiss Axioscope A1 microscope equipped with four fluorescence channels widefield IF. Microphotographs were collected using a Zeiss Axiocam 503 Color digital camera with the Zen 2.0 Software (Zeiss).

### Statistical Analysis

Means and standard deviation were calculated for continuous variables, whereas frequencies and percentages were reported for categorical variables. Non-parametric data were assessed by Mann-Whitney U-tests. Patient data (**Table 1**) were analyzed by T-Student test. Data from *in vivo* mouse models were analyzed using two-way analysis of variance (ANOVA). Results were expressed as mean ± standard deviations. and P-values <0.05 were considered statistically significant. All statistical analyses were performed using GraphPad Prism software 9.0 (GraphPad Software Inc., La Jolla, CA, USA).

## Results

### Analysis of Placental Deposition of Early C Components

**Figure 1:**
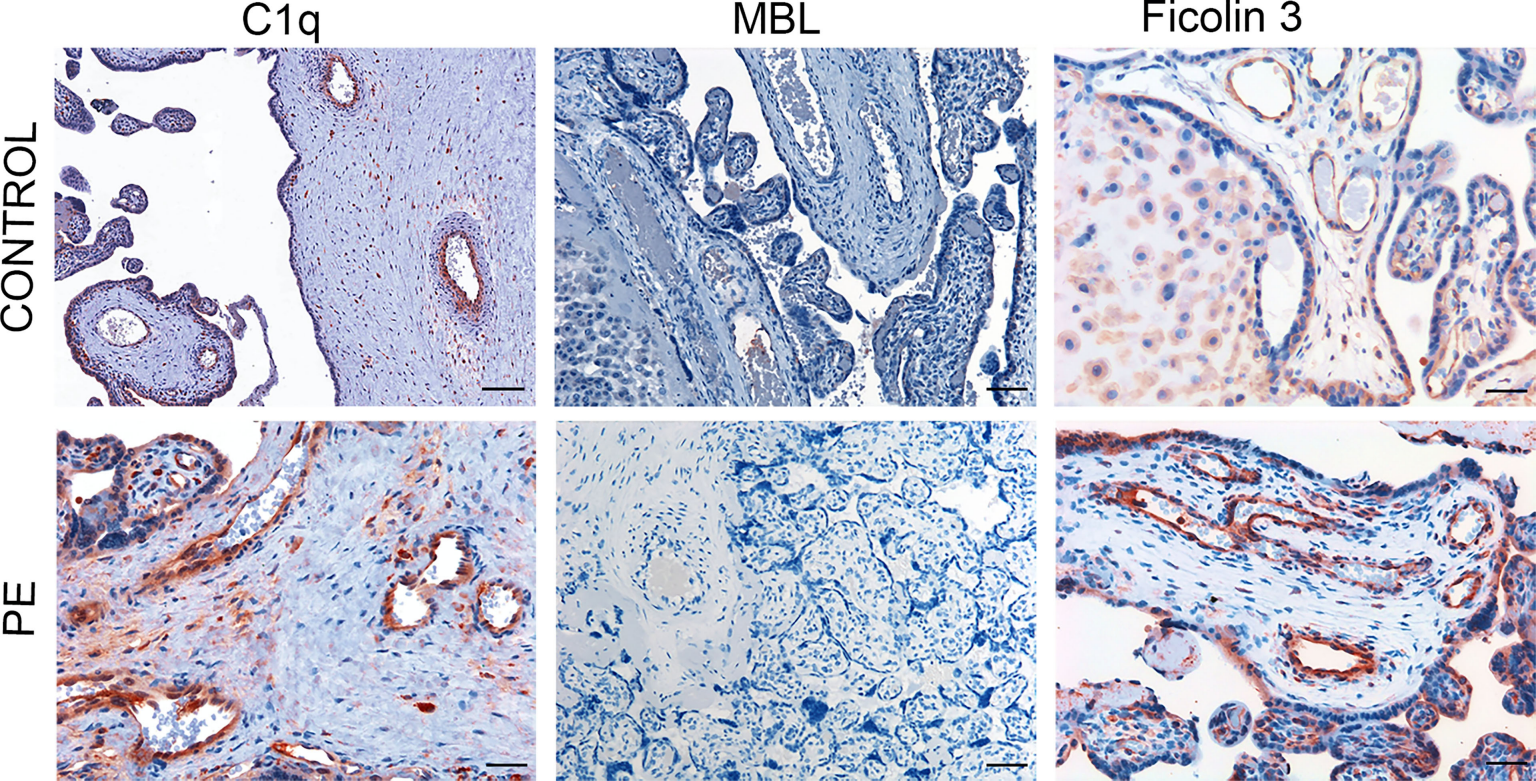
Immunohistochemical analysis of initiators of the classical and lectin complement pathways in placental tissue. Sequential sections of pre-eclamptic (PE) and normal (CONTROL) placentae were stained for C1q, MBL and ficolin-3. The panel presents representative images from 15 early-onset PE patients and 15 controls showing localization of C1q in the decidual vessels of both control and early-onset PE placentae with more intense staining in the latter. Deposits of ficolin-3 were seen almost exclusively in decidual vessels of PE placenta while MBL was undetectable. Scale bars, 50 μm.

To elucidate the mechanism of C activation in PE placentae, we searched for the presence and distribution of C components that may initiate C activation through the classical and/or the lectin pathway. C1q was detected in both control and PE placentae, though with different staining intensity. A detailed analysis of normal placentae showed that C1q was localized on vascular endothelium and stroma of decidua, while virtually undetectable in chorionic villi (**Figure 1**). The distribution pattern of C1q staining in PE placentae was similar to that of control placentae with the only difference that the staining was more intense in decidua and was also seen on syncytiotrophoblasts of some villi (**Figure 1**). We also examined the placentae for the presence of the initiators of the C lectin pathway and found that MBL was undetectable in both normal and pathological tissue samples (**Figure 1**) while clearly documented in the liver (**Supplemental Figure 1A**). Conversely, deposits of ficolin-3 were observed on decidual vascular endothelium and syncytiotrophoblasts of PE placentae. The staining was weak in 25% and more intense in 75% of PE placentae, while slightly detectable in the control samples (**Figure 1**). The anti-ficolin-1 antibody reacted almost exclusively with syncytiotrophoblasts of normal and PE samples with no significant difference in the staining intensity between the two groups of placentae (**Supplemental Figure 2**), while ficolin-2 was practically undetectable.

### Placental Deposition of C4 Convertases

**Figure 2:**
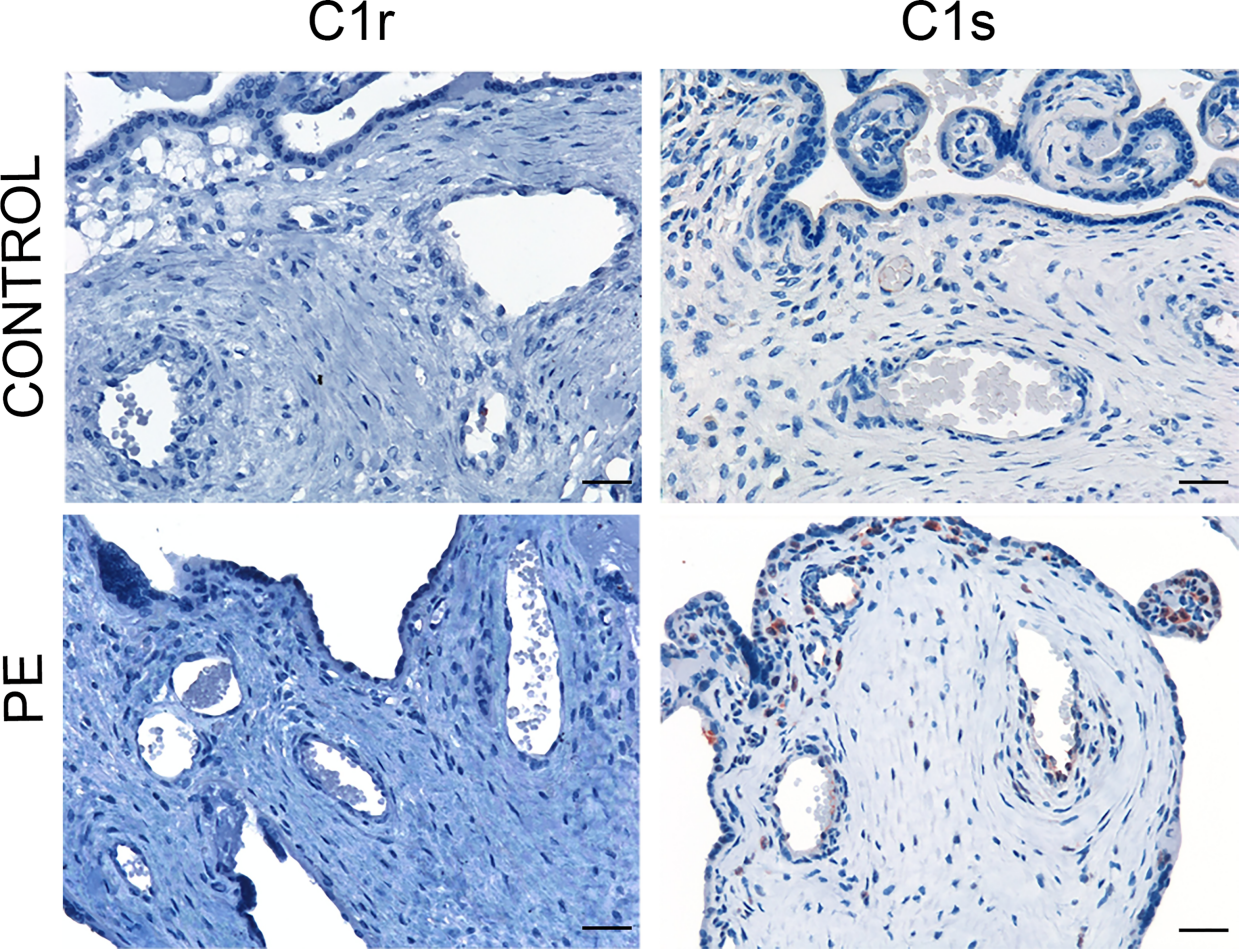
Immunohistochemical analysis of C4 convertases of the classical complement pathway in placental tissue. Sections of pre-eclamptic (PE) and normal (CONTROL) placentae were stained for C1r and C1s. The panel shows representative images of PE and control placentae documenting complete absence of these C components in both groups of placentae. Scale bars, 50 μm.

**Figure 3:**
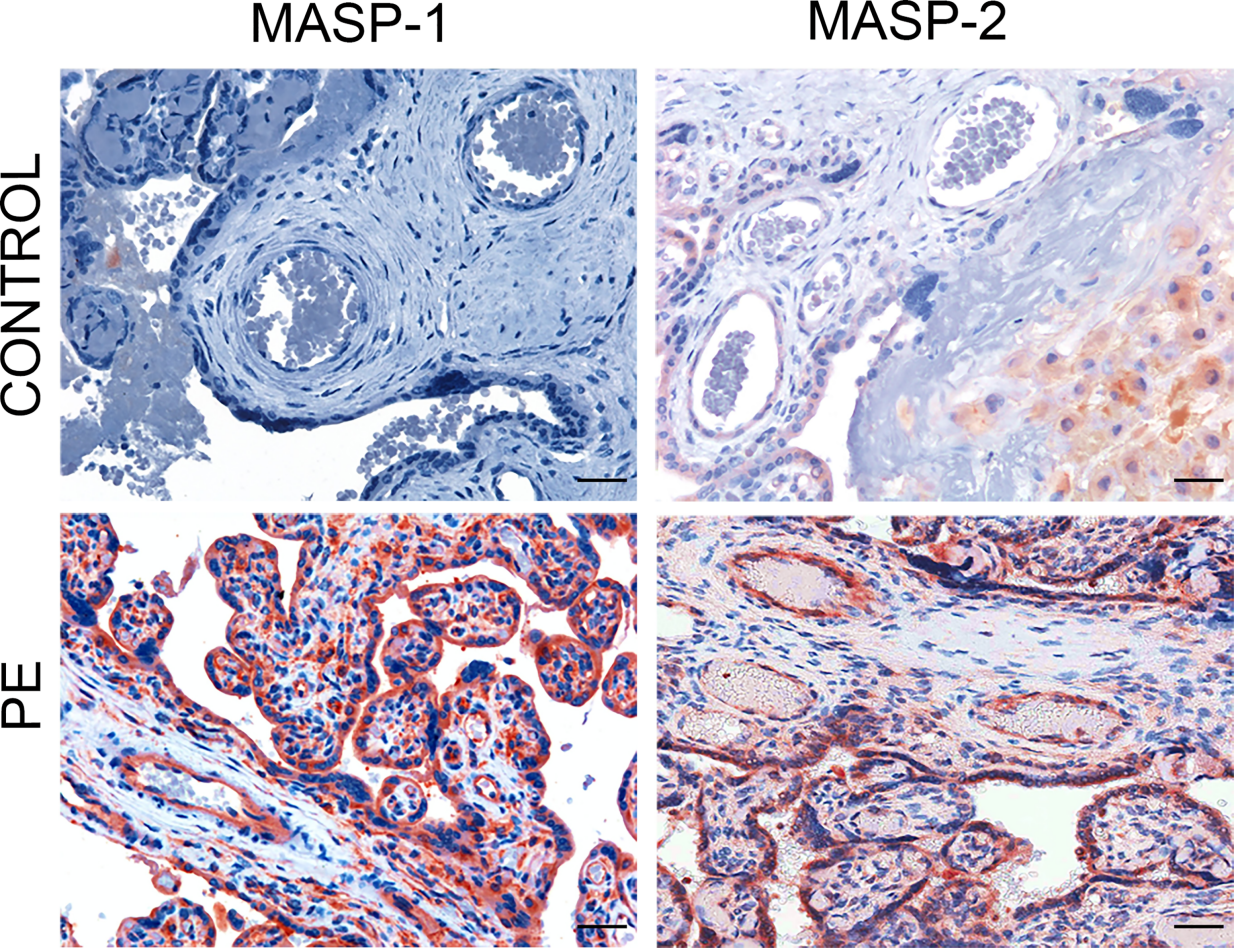
Immunohistochemical analysis of C4 convertases of the lectin complement pathways in placental tissue. Sections of pre-eclamptic (PE) and normal (CONTROL) placentae were stained for MASP-1 and MASP-2. The panel shows representative images of MASP-1 and MASP-2 deposits on decidual vessels, syncytiotrophoblasts and villous microvessels of PE placentae that were not seen in control placentae. Scale bars, 50 μm.

C activation was investigated by analyzing the presence of C4 convertases of the classical and lectin pathways. Despite the substantial deposits of C1q, we failed to reveal C1r and C1s in normal and PE placentae (**Figure 2**) using antibodies that were able to detect intracellular C1r and C1s in hepatocytes of paraffin-embedded liver tissue (**Supplemental Figures 1C, D**). Conversely, MASP-1 and MASP-2 exhibited a characteristic distribution pattern with a diffuse localization on decidual endothelial cells, syncytiotrophasts and villous microvessels of PE placentae but absent in normal controls (**Figure 3**). A substantial proportion of PE placentae were positive for MASP-1 (56%) and MASP-2 (87%) and the remaining placentae were weakly positive.

### Deposition of C Activation Productsin Placenta

**Figure 4:**
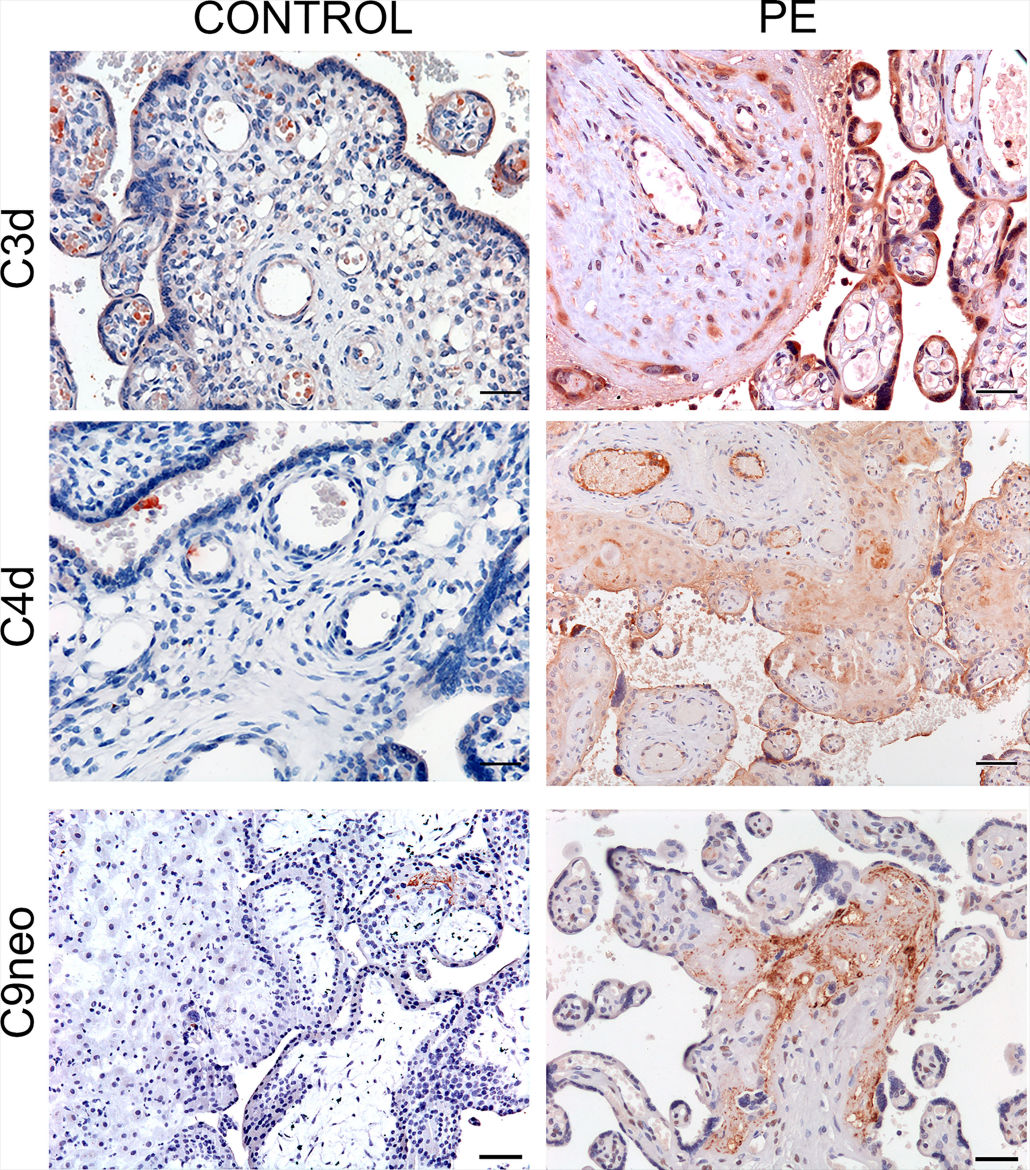
Immunohistochemical analysis of C activation products in placental tissue. Sequential sections of pre-eclamptic (PE) and normal (CONTROL) placental tissue were stained for C4d, C3d and C9 neo. The panel shows representative images revealing localization of the C activation products in the decidual vessels and in some villi of PE placentae and their absence in the control tissue. Scale bars, 50 μm.

As the presence of MASP-1 and MASP-2 deposition indicates that C activation may proceed through the lectin pathway, we stained the placental tissue for C4d and C3d that play a central role in the C cascade. Both C activation products were found in the decidual blood vessels and villous trophoblasts of 50% and 56% PE placentae respectively and were weakly positive in all the other pathologic placentae while hardly detectable in the control tissue samples (**Figure 4**). To further ascertain if the terminal pathway was also activated in PE placentae, the tissue samples were examined for the deposition of the terminal C complex C5b-9 using an antibody that detects a neoantigen of C9 (C9 neo) present in the assembled complex (39). Staining for C5b-9 complex was positive in 45% and weakly positive in 55% of PE placentae and was mainly observed in decidual vessels, but was absent in control placentae (**Figure 4**).

### C1q Expression in Perivascular Extravillous Trophoblasts

**Figure 5:**
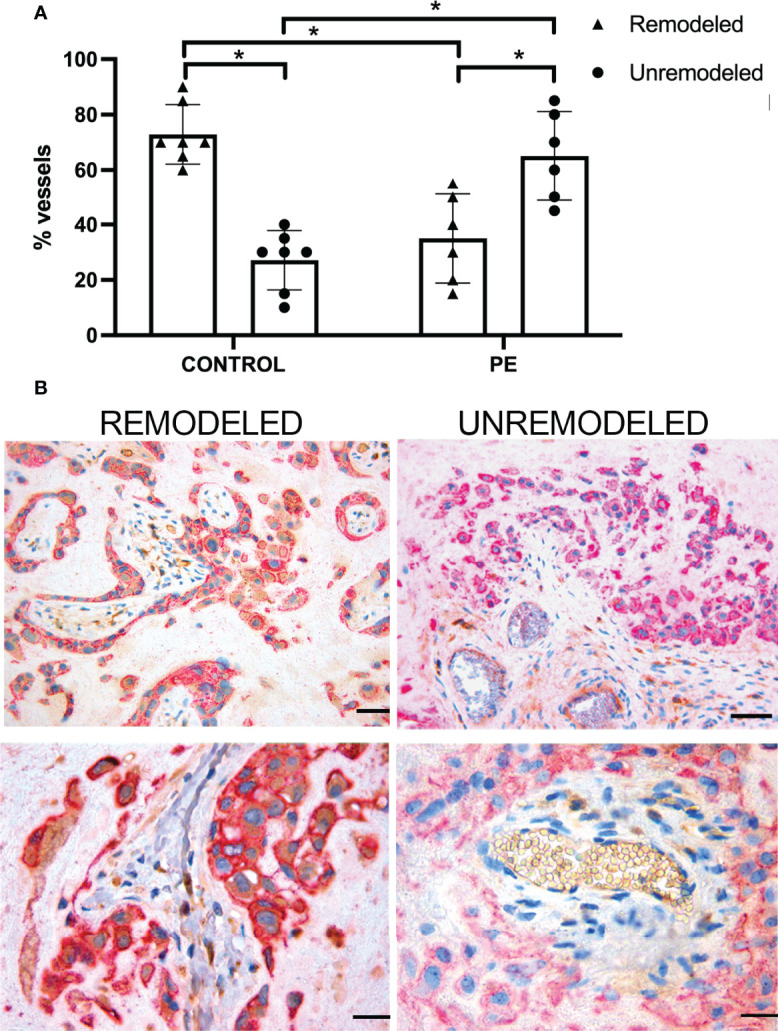
Vascular remodeling in human placentae. Tissue sections were stained with hematoxylin and eosin and examined for the number of remodeled and unremodeled vessels in placental decidua. **(A)** The figure shows the percentage (± SD) of the two types of decidual vessels found in sections of normal (n = 7, CONTROL) and pre-eclamptic (n = 6, PE) placentae. After Mann-Whitney test add (*p < 0.001). **(B)** Representative images of placental sections double stained for C1q (brown) and cytokeratin 7 (red) expressed in trophoblasts. Note the presence of C1q in trophoblast surrounding remodeled vessels (upper and lower left panels) and its absence in trophoblasts surrounding unremodeled vessels (upper and lower right panels). Scale bars, 50 μm.

Following our previous observations that extravillous trophoblasts (EVT) acquire the ability to synthesize and secrete C1q used by these cells to invade the decidua (31), we sought to investigate the distribution of EVT expressing C1q in PE and control decidua and to determine their contribution to vascular remodeling. The extent of arterial changes was evaluated by examining the disruption of the muscle cells of tunica media in decidual vessels recognized by an antibody against α-smooth muscle actin (α-SMA). **Supplemental Figure 3** shows decidual vessels of a PE placenta with different degrees of vascular remodeling assessed by the decrease in the width of the muscle cell layer. Over 70% (72.86%) of decidual vessels in normal pregnancy undergo remodeling as opposed to 27% of unremodeled vessels, while this ratio is inverted in PE placentae with a prevalence of vessels that remain unremodeled (**Figure 5A**). PE placental sections were double-stained for C1q and cytokeratin 7 (CK7) to identify C1q-containing trophoblasts. Analysis of perivascular trophoblasts revealed the presence of C1q in cells surrounding remodeled vessels, in contrast to trophoblasts localized around unremodeled vessels that failed to express this C component (**Figure 5B**). The distribution pattern of trophoblasts with or without C1q around remodeled and unremodeled vessels, respectively, was similar in PE and control placentae. Interestingly, the absence of C1q was restricted to perivascular trophoblasts and did not involve interstitial trophoblasts migrating at some distance from spiral arteries that invariably contained C1q (**Supplemental Figure 4**).

### Vascular Remodeling in Implantation Sites of *C1qa^+/+^*, *C1qa^+/-^* and *C1qa^-/-^* Mice

**Figure 6:**
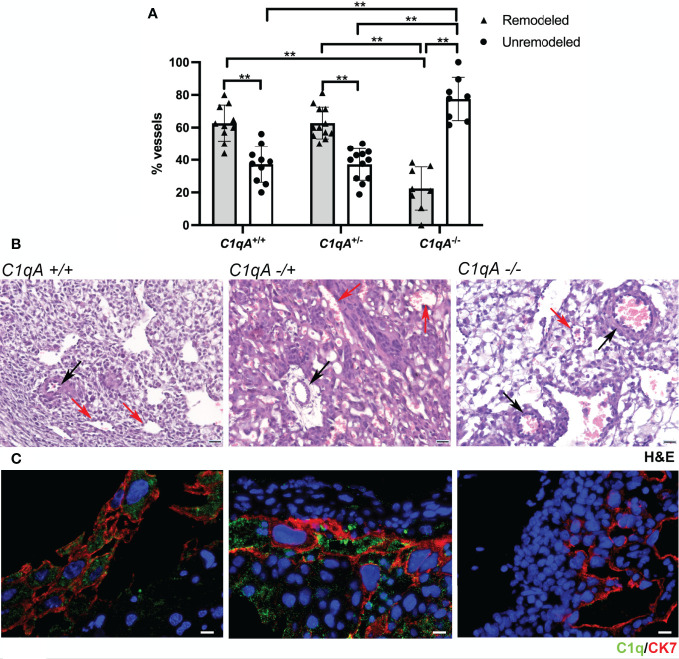
Vascular remodeling in murine placentae. Tissue sections were stained with hematoxylin and eosin and examined for the number of remodeled and unremodeled vessels in placental decidua. **(A)** The upper panel shows the percentage (± SD) of the two types of decidual vessels found in embryo implantation sites from WT *C1qa^+/+^* (n = 10), heterozygous *C1qa^+/-^* (n = 12) and *C1qa^-/-^* (n = 8) mice. After (ANOVA) add (**p < 0.0001). **(B)** The middle panel shows representative sections of implantation sites from *C1qa^+/+^*, *C1qa^+/-^* and *C1qa^-/-^* mice containing remodeled (red arrow) and unremodeled (black arrow) vessels. Scale bar, 50 μm. **(C)** The lower panel shows representative immunofluorescence images from sections of implantation sites from *C1qa^+/+^*, *C1qa^+/-^* and *C1qa^-/-^* mice stained for C1q (green) and CK7 (red). Note the presence of C1q only in CK7 positive trophoblasts from *C1qa^+/+^* and *C1qa^+/-^* mice. Scale bars, 50 μm.

We have previously reported reduced labyrinth development, decreased trophoblast invasion, and impaired vascular remodeling in the implantation site of pregnant *C1qa^-/-^* mice compared to WT animals of the same gestational age (31). To further investigate if trophoblasts synthesizing C1q are directly involved in the process of vascular remodeling, we examined the decidua of female *C1qa^-/-^* mice mated with either C1q sufficient or C1q deficient male mice and compared the results with those observed in the decidua of WT mice. As shown in **Figure 6A**, the percentage of decidual remodeled vessels in WT mice was significantly higher than that of unchanged vessels, whereas unremodeled vessels were prevalent in *C1qa^-/-^* mice. Interestingly, the percentages of changed and unchanged vessels in heterozygous *C1qa^+/-^* mice were essentially similar to those observed in WT mice (**Figure 6A**). Representative sections of placenta from WT, heterozygous and C1q deficient mice showing the distribution of remodeled and unremodeled vessels are presented in **Figure 6B**. Staining of placental sections from the three groups of mice revealed the presence of C1q in trophoblasts of both WT and heterozygous mice and the expected absence of C1q expression in C1q deficient mice (**Figure 6C**).

## Discussion

Previous studies have documented C deposits on chorionic villi and in particular on villous trophoblasts of PE placentae, suggesting the contribution of C to the development of placental alterations in PE (21–25). We have now extended the C analysis to the decidual tissue based on the understanding that the pathologic process starts in the decidua involving primarily the vessels and subsequently affects the villi. The data presented in this study show that C is activated in PE placenta through the lectin pathway and that the role of C1q is to promote vascular remodeling in decidua rather than to activate the C classical pathway.

Examination of the placentae from healthy controls revealed a negligible presence of C components, most likely due to an inflammatory-like process that develops in placental decidua as a result of tissue remodeling associated with embryo implantation (8, 9). The only exception to this general rule is the diffuse deposition of C1q on decidual vessels and stroma in all placental samples examined. This is consistent with our previous findings that C1q is synthesized and secreted by endothelial cells and extravillous trophoblasts in the decidua in normal pregnancy (30, 31). The increased expression of C1q observed in decidua and on the surface of villi in the PE patients may be attributed to the binding of C1q to endothelial cells and trophoblasts as a result of cellular changes that occur in PE.

Different from C1q the other C components and the C activation products were focally distributed on decidual vessels and villous trophoblasts and were most likely deposited in areas of histopathologic alterations of the placental tissue. The localization of C4d and C3d in PE placenta is a clear indication of C activation that may reach completion with the deposition of terminal C complex revealed by a monoclonal antibody directed against a neoantigen of C9 expressed in C5b-9 complex (39). Linear deposits of C4d on syncytiotrophoblasts were reported to be associated with lower gestational age and fetal growth restriction in PE patients suggesting the contribution of C activation to the development of these clinical manifestations (23, 25). However, it is unclear whether C activation is a secondary event triggered by cells undergoing apoptosis or is directly responsible for trophoblast damage. It should be pointed out that syncytrophoblasts are relatively resistant to C-mediated killing, which requires the presence of cytotoxic antibodies and a marked decrease in the expression of the C regulators CD46 and CD59 (40). C dependent cytotoxic antibodies are infrequently found in PE patients except for those with antiphospholipid syndrome associated with PE (41). In addition, C regulators have been reported to be normally expressed both at molecular and protein levels in the placenta of PE patients (23, 24). However, we cannot exclude a sublytic effect of C on syncytiotrophoblasts resulting in the release of microparticles that circulate in maternal blood in normal pregnancy and at increased level in PE patients (42).

Most likely C activation plays a more critical role in decidua promoting inflammation and stimulating a hypercoagulable state that are both features of maternal abnormalities implicated in the second stage of PE pathogenesis (43). In this context C5a and C5b-9 have a significant impact on vascular endothelium by inducing release of proinflammatory cytokines, enhancing the expression of tissue factor and increasing vascular permeability (44). Whatever the mechanism involved in C-mediated placental damage in human preeclampsia, C activation has been shown to play an essential role in a mouse model of PE causing adverse pregnancy outcomes that can be prevented by the administration of C inhibitors (28, 32, 45).

The co-localization of C1q and C4 on syncytiotrophoblasts of PE placentae in the absence of MBL led Buurma and colleagues (23) to suggest that C is activated *via* the classical pathway possibly triggered by the binding of antibodies, although the association between C4d and immune complex deposits was not statistically significant. Our failure to detect C1r and C1s deposition at sites of C1q and C4d deposition in this study rules out the direct activation of the classical pathway and favors the activation of the lectin pathway. Consistent with this is our previous report that pregnancy loss in a mouse model of PE is mediated by MBL-dependent activation of the lectin pathway (32). However, we could not detect MBL in human PE placentae indicating that the results obtained in mice do not fully replicate the observations in PE patients as suggested by the different molecular organization of human and mouse MBL. Although mice and higher primates possess two *MBL* genes, *MBL1* and *MBL2*, only one *MBL* gene (*MBL2*) give rise to protein in man, while *MBL1* is expressed as pseudogene (*MBL1P1*) (46). By contrast, the two forms of MBL in mice are encoded by two distinct functional genes, known as *mbl-a* and *mbl-c*, and, consequently, the protein concentration of mouse MBL may be 10-fold higher than that of human MBL. Another important point to consider is that ficolin-3, the most abundant and most potent lectin pathway activator in man, is not present in mice, making a direct comparison of the lectin pathway activity between mice and humans difficult (47). Thus, the absence of MBL does not exclude activation of the lectin pathway by other initiators including ficolins and collectins as suggested by the strong staining of decidual vessels and villous syncytiotrophoblasts for ficolin-3, MASP-1, and MASP-2. The finding of ficolin-3 deposited on villous syncytiotrophoblasts of PE placentae is in agreement with the observations by Wang and colleagues (48), who reported binding of ficolins to cells undergoing apoptosis and suggested that they may contribute to promote local inflammatory responses. While we are not excluding this possibility, our data showing deposition of MASPs and other C components on syncytiotrophoblasts indicate that ficolin-3 most probably acts as trigger of the lectin pathway. Ficolin-3-mediated C activation is likely to plays an important role in decidual vascular endothelium by contributing to endothelial activation and dysfunction as documented by the increased levels of endothelial-derived factors observed in PE patients, including inflammatory cytokines (6) and soluble adhesion molecules (49). Based on our results, we would speculate that the ficolin-3 gene, *FCN3*, may be turned on locally, particularly in the vascular bed, in response to different stimuli (50) and contributes to the pathophysiologic mechanisms causing PE in humans, a process that cannot be replicated in murine models (51). The subarachnoid hemorrhage in humans is another example of human pathologic condition associated with ficolin-3-dependent C activation (52).

We have previously provided evidence for an alternative role of C1q in normal pregnancy by showing impaired remodeling of the decidual vessel in C1q deficient pregnant mice (31), a recognized animal model of PE (27), and reduced levels of C1q mRNA transcripts in PE placental tissue (53). The finding that the unremodeled decidual vessels more frequently observed in PE patients are surrounded by trophoblasts that do not stain for C1q indicates that the vascular changes are associated with the expression of C1q in these cells. This is consistent with the observation that the defective remodeling of blood vessels in C1q deficient pregnant mice can be reversed by mating these animals with C1q sufficient mice, resulting in an increased number of remodeled vessels and the perivascular appearance of C1q-expressing trophoblasts. The EVT that invade the decidua in heterozygous and normal animals share the ability to synthesize C1q. The decreased and restricted expression of C1q to perivascular trophoblasts in decidual EVT from PE patients could be explained by the special microenvironment organized around the remodeled vessels that helps C1q-containing EVT to exert disruptive activity on the vascular wall (54). The mechanisms by which C1q modulates EVT effector functions remain to be defined.

In conclusion, we have shown that C components and C activation products are deposited on placental tissue obtained from PE patients but are absent in the control placentae except for C1q that is constitutively expressed at the placental level in physiological pregnancy. Our data support the notion that in human PE C is locally activated through the lectin pathway triggered by ficolin-3 whilst MBL does not appear to be involved. Equally, C1q is not implicated in the activation of the classical C pathway and should be considered a biomarker of EVTs that contribute to decidual vascular remodeling.

## Data Availability Statement

The original contributions presented in the study are included in the article/**Supplementary Material**. Further inquiries can be directed to the corresponding author.

## Ethics Statement

The study was reviewed and approved by the Regional Ethical Committee of FVG (CEUR), Udine, Italy (CEUR-2020-Os-156; Prot. 0022668/P/GEN/ARCS). The patients/participants provided their written informed consent to participate in this study. The animal study was reviewed and approved by The UK Home Office.

## Author Contributions

FT, BB, and AM designed the study, analyzed the results, and wrote the manuscript. PG, MB, and RB provided resources, expertise and critically reviewed the manuscript. AG, VC, and GM performed the immunohistochemical staining. GR and FF organized patient recruitment. AM and CA prepared figures and tables and edited the manuscript. All authors contributed to the article and approved the submitted version.

## Funding

This research was supported by grants from the Institute for Maternal and Child Health, IRCCS Burlo Garofolo, Trieste, Italy (RC20/16, RC23/18, 24/19 to G.R. and RC09/21 to CA).

## Conflict of Interest

The authors declare that the research was conducted in the absence of any commercial or financial relationships that could be construed as a potential conflict of interest.

## Publisher’s Note

All claims expressed in this article are solely those of the authors and do not necessarily represent those of their affiliated organizations, or those of the publisher, the editors and the reviewers. Any product that may be evaluated in this article, or claim that may be made by its manufacturer, is not guaranteed or endorsed by the publisher.

## Supplementary Material

Supplementary Figure 1 Immunohistochemical analysis of MBL, ficolin-2, C1r and C1s in liver tissue. Liver sections were stained for MBL **(A)**, ficolin-2 **(B)**, C1r **(C)** or C1s **(D)** as positive control for these C components that were undetectable in PE placentae. Scale bars, 50 μm.

Supplementary Figure 2 Immunohistochemical analysis of ficolin-1 in placental tissue. Sections of pre-eclamptic (PE) and normal (CONTROL) placentae were stained for ficolin-1. The panel shows representative images of ficolin-1 deposits mainly localized on syncytiotrophoblasts of both PE and control placentae. Scale bars, 50 μm.

Supplementary Figure 3 Analysis of vascular changes and trophoblast distribution in pre-eclamptic human placenta. The sections were stained anti-α-SMA antibody (brown) to reveal smooth muscle cells and CK7 (red) to document the presence of trophoblasts. A high proportion of vessels (arterioles) showed a preserved structure of the tunica media (upper and lower left panels). Along with preserved arterioles, vessels with various degrees of remodeling were also observed (upper and lower right panels). At sites of vascular remodeling trophoblast cells were detected in close proximity with the vessels. Scale bars, 50 μm.

Supplementary Figure 4 C1q expression in interstitial trophoblast. Representative section of PE placenta double-stained for C1q (brown) and CK7 (red). The images show CK7 positive trophoblasts (red arrows) infiltrating the decidua at some distance from blood vessels and express C1q. Scale bars, 50 μm.
