# Supplementary figures and images for: Distinct Roles of Classical and Lectin Pathways of Complement in Preeclamptic Placentae
Source: Front Immunol. 2022 May 31;13:882298. doi: 10.3389/fimmu.2022.882298 (PMC9197446; doi:10.3389/fimmu.2022.882298)

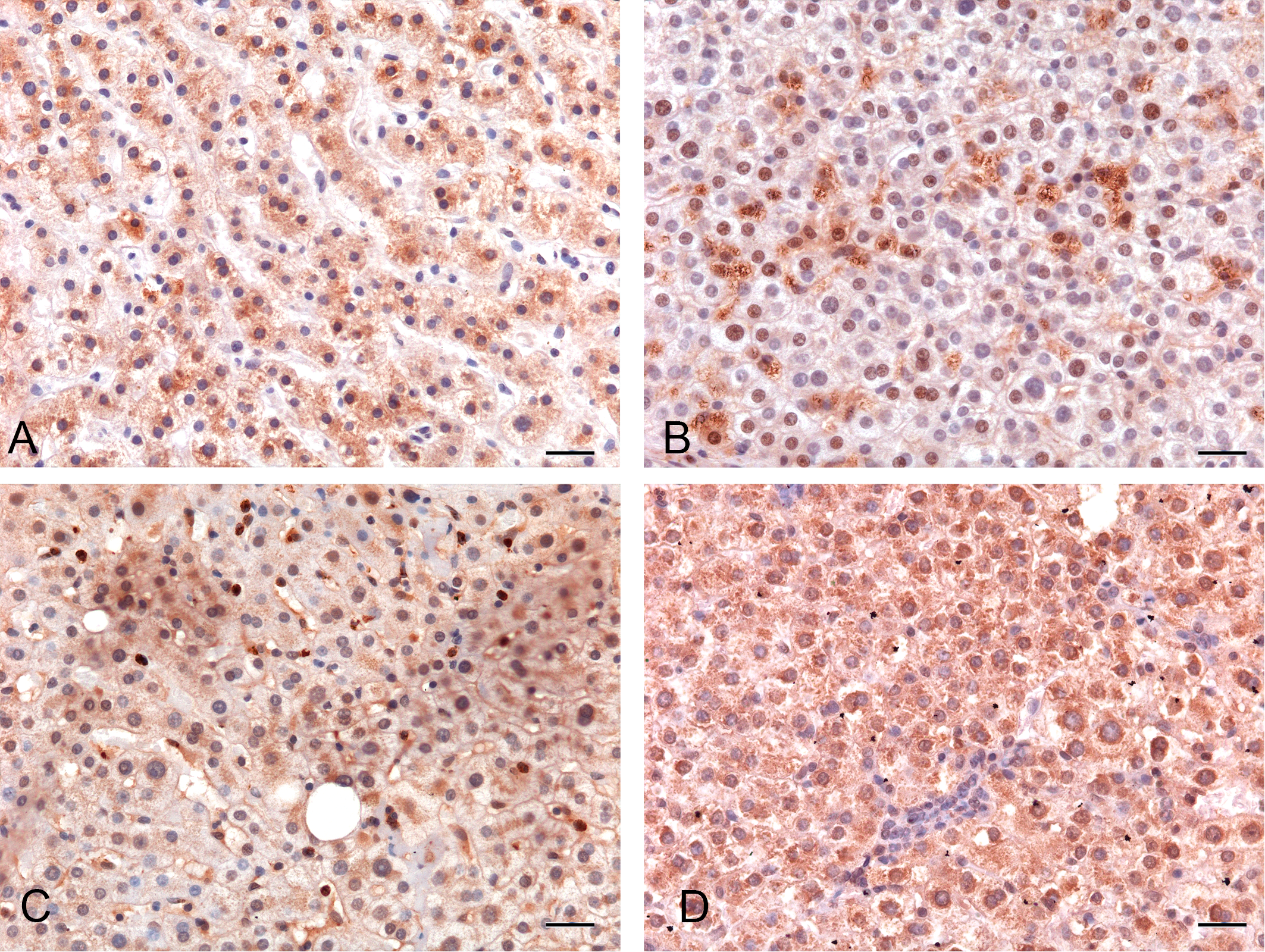

Supplement: Supplementary Figure 1 — Immunohistochemical analysis of MBL, ficolin-2, C1r and C1s in liver tissue. Liver sections were stained for MBL (A), ficolin-2 (B), C1r (C) or C1s (D) as positive control for these C components that were undetectable in PE placentae. Scale bars, 50 μm. [file Image_1.tif]

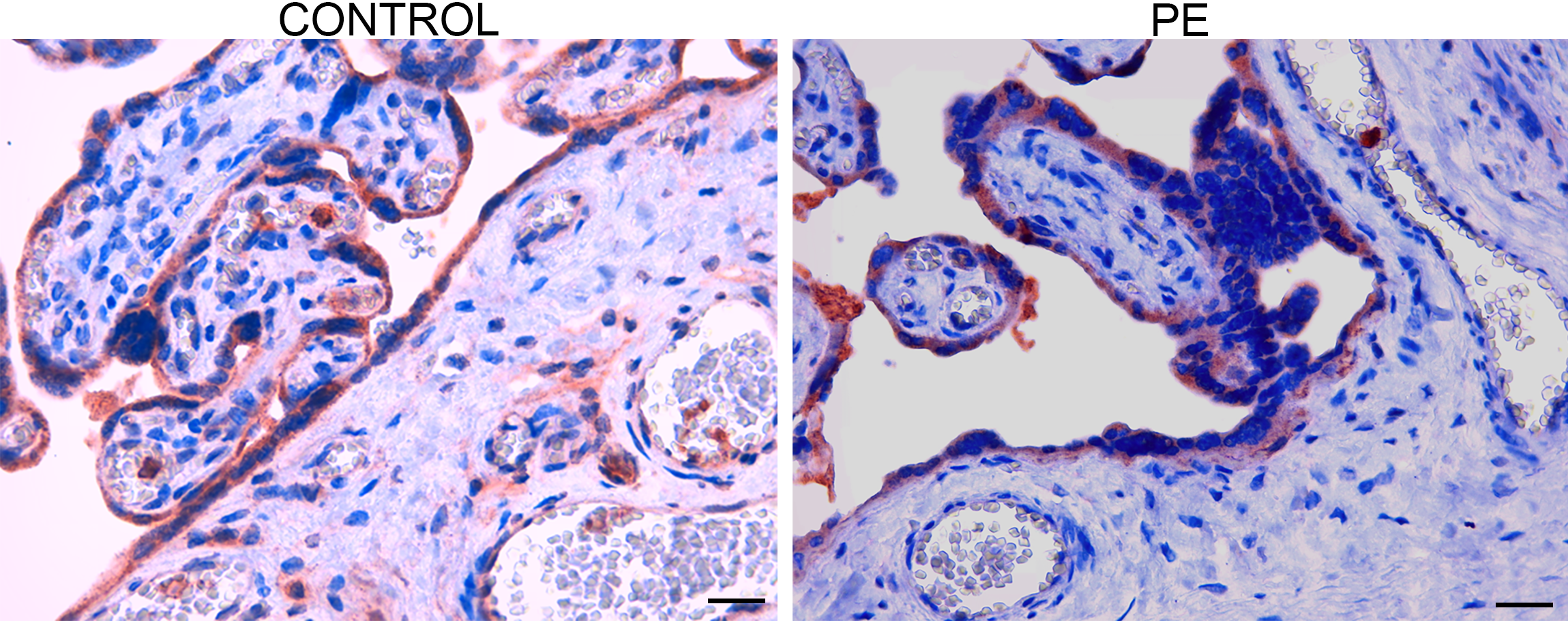

Supplement: Supplementary Figure 2 — Immunohistochemical analysis of ficolin-1 in placental tissue. Sections of pre-eclamptic (PE) and normal (CONTROL) placentae were stained for ficolin-1. The panel shows representative images of ficolin-1 deposits mainly localized on syncytiotrophoblasts of both PE and control placentae. Scale bars, 50 μm. [file Image_2.tif]

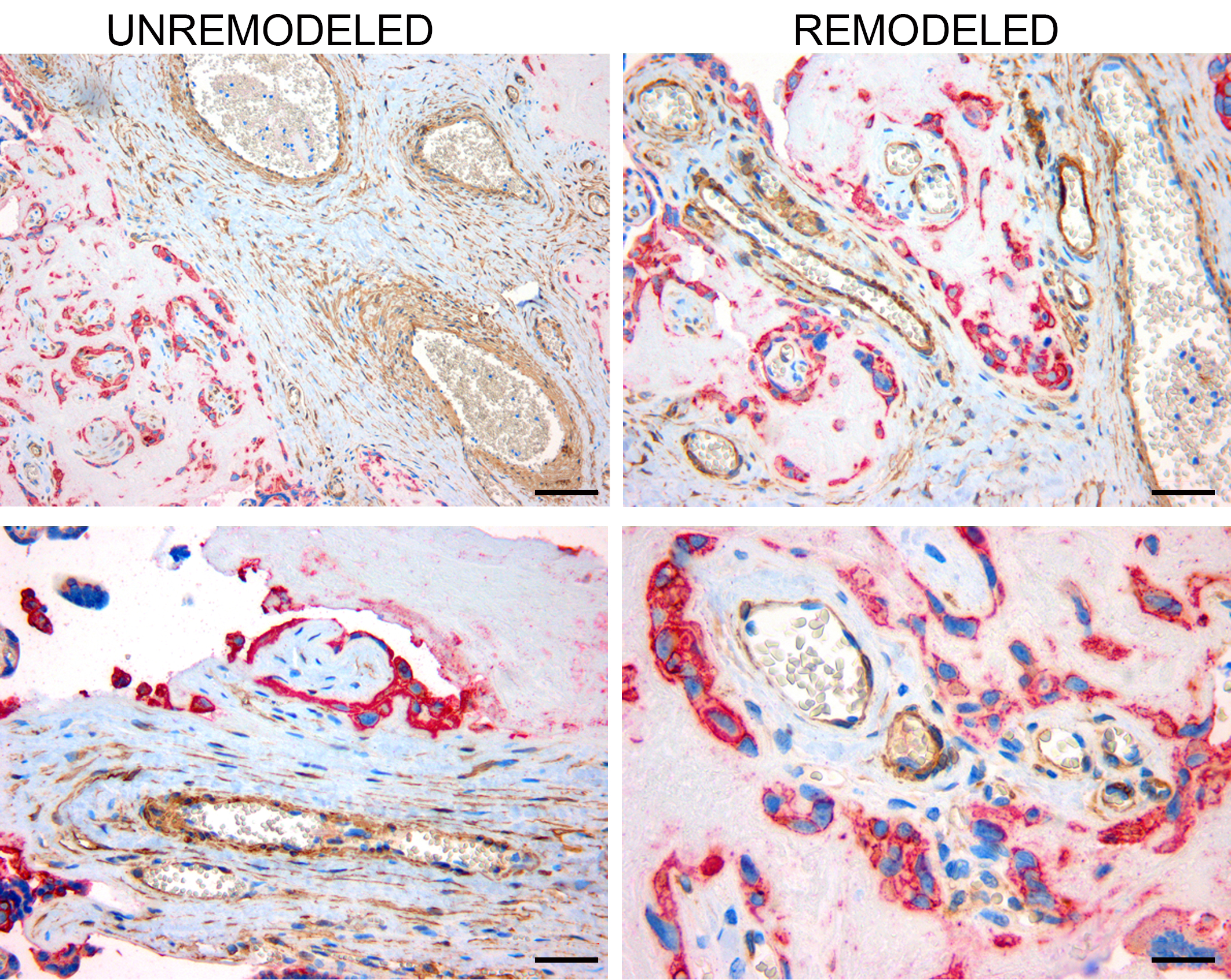

Supplement: Supplementary Figure 3 — Analysis of vascular changes and trophoblast distribution in pre-eclamptic human placenta. The sections were stained anti-α-SMA antibody (brown) to reveal smooth muscle cells and CK7 (red) to document the presence of trophoblasts. A high proportion of vessels (arterioles) showed a preserved structure of the tunica media (upper and lower left panels). Along with preserved arterioles, vessels with various degrees of remodeling were also observed (upper and lower right panels). At sites of vascular remodeling trophoblast cells were detected in close proximity with the vessels. Scale bars, 50 μm. [file Image_3.jpg]

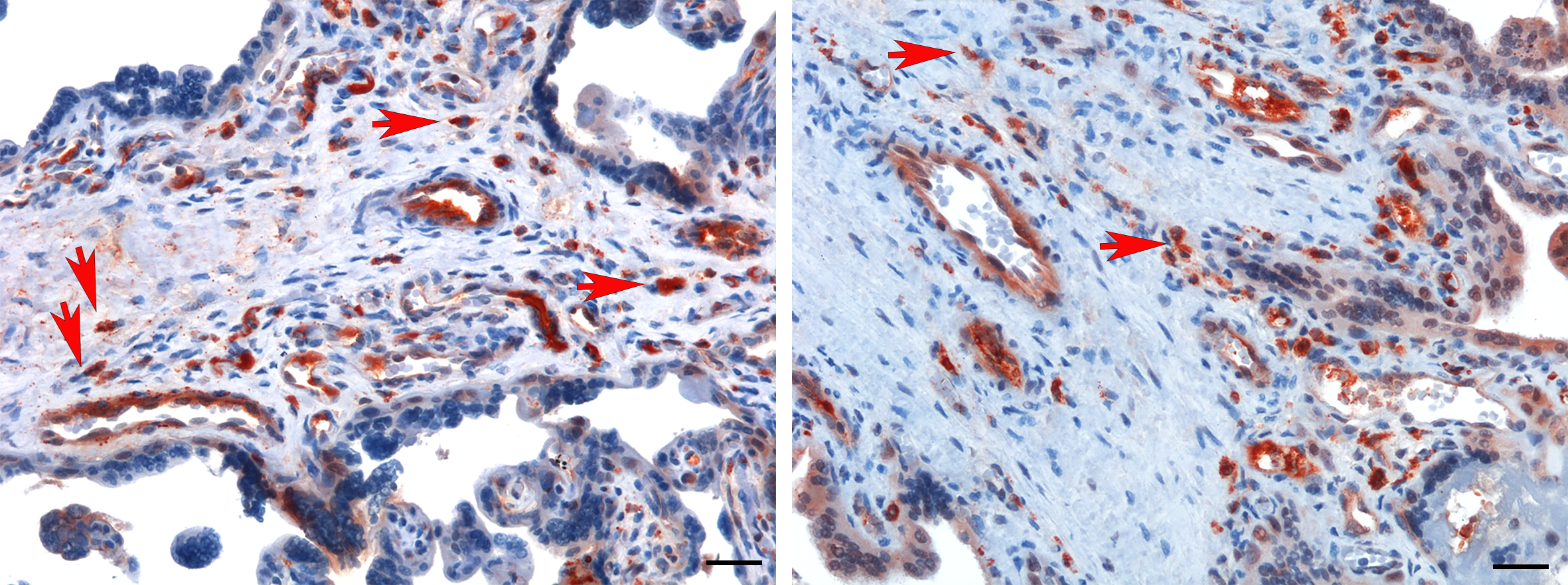

Supplement: Supplementary Figure 4 — C1q expression in interstitial trophoblast. Representative section of PE placenta double-stained for C1q (brown) and CK7 (red). The images show CK7 positive trophoblasts (red arrows) infiltrating the decidua at some distance from blood vessels and express C1q. Scale bars, 50 μm. [file Image_4.tif]
